# Urinary expression of let-7c cluster as non-invasive tool to assess the risk of disease progression in patients with high grade non-muscle invasive bladder Cancer: a pilot study

**DOI:** 10.1186/s13046-020-01550-w

**Published:** 2020-04-17

**Authors:** Manuela Spagnuolo, Manuela Costantini, Mariaconsiglia Ferriero, Marco Varmi, Isabella Sperduti, Giulia Regazzo, Lucia Cicchillitti, Ana Belén Díaz Méndez, Giovanni Cigliana, Vincenzo Pompeo, Andrea Russo, Valentina Laquintana, Riccardo Mastroianni, Giulia Piaggio, Umberto Anceschi, Aldo Brassetti, Alfredo Bove, Gabriele Tuderti, Rocco Simone Flammia, Michele Gallucci, Giuseppe Simone, Maria Giulia Rizzo

**Affiliations:** 1 Department of Research, Advanced Diagnostics and Technological Innovation, Genomic and Epigenetic Unit, Translational Research Area, IRCCS Regina Elena National Cancer Institute, Via Elio Chianesi 53, 00144 Rome, Italy; 2 Department of Experimental Clinical Oncology, Urology Unit, IRCCS Regina Elena National Cancer Institute, Via Chianesi 53, 00144 Rome, Italy; 3 Biostatistical Unit, IRCCS Regina Elena National Cancer Institute, Via Chianesi 53, 00144 Rome, Italy; 4 Department of Experimental Clinical Oncology, Gynecologic Oncology Unit, IRCCS Regina Elena National Cancer Institute, Via Chianesi 53, 00144 Rome, Italy; 5 Department of Research, Advanced Diagnostics and Technological Innovation, Clinical Pathology Unit, IRCCS Regina Elena National Cancer Institute, Via Chianesi 53, 00144 Rome, Italy; 6 Department of Research, Advanced Diagnostics and Technological Innovation, Pathology Unit, IRCCS Regina Elena National Cancer Institute, Via Chianesi 53, 00144 Rome, Italy; 7 Department of Research, Advanced Diagnostics and Technological Innovation, SAFU Unit, Translational Research Area, IRCCS Regina Elena National Cancer Institute, Via Chianesi 53, 00144 Rome, Italy; 8 Department of Urology, “Sapienza” University, Rome, Italy; 9 Department of Clinical and Experimental Oncology, IRCCS Regina Elena National Cancer Institute, Via Elio Chianesi 53, 00144 Rome, Italy

**Keywords:** Non-muscle-invasive bladder cancer, microRNA, Let-7c cluster, urinary biomarkers, progression free survival

## Abstract

**Background:**

High grade non-muscle-invasive bladder cancer (HG-NMIBC) is a heterogeneous disease with variable risk of progression. Urinary microRNAs are promising biomarkers for BC detection and surveillance. Let-7c-5p miRNA, clustered with miR-99a-5p and -125b-5p, is deregulated in cancer, including BC. The aim of this study is to evaluate urinary let-7c cluster expression in Ta/T1 HG-NMIBC patients and its impact on progression-free survival (PFS).

**Methods:**

Quantitative Real-Time-Polymerase-Chain-Reaction (qRT-PCR) was used to analyze the let-7c cluster expression in 57 urine and 49 neoplastic paired tissue samples prospectively collected from transurethral resection (TUR) HG-NMIBC patients. Twenty urine and 10 bladder tissue samples were collected and analyzed as normal controls. QRT-PCR was also used to detect intra−/extra-cellular let-7c cluster in BC cells. Receiver Operating Characteristic (ROC) curves were used to identify urinary miRNAs cut-off values predicting T-stage and PFS. Uni/multivariable Cox regression was performed to identify predictors of PFS. A nomogram predicting progression risk and a decision curve analysis (DCA) were performed.

**Results:**

Urinary let-7c was significantly up-regulated in patients compared with controls, while the whole cluster was down-regulated in tumor tissues. Supporting these findings, in vitro comparison of extra−/intra-cellular ratios of cluster levels between BC cells, showed a higher ratio for let-7c in HG-NMIBC versus low-grade cells. Urinary let-7c cluster expression was increased in higher T-stage and was an independent predictor of progression. Lower EORTC-score and downregulation of urinary cluster were predictors of higher PFS on univariable Cox regression, while on multivariable analysis only cluster expression was an independent progression predictor. On DCA, a benefit was evident for patients with a PFS probability > 20%.

**Conclusions:**

Urinary let-7c cluster evaluation may improve prognosis, identifying patients at risk of progression and addressing early radical treatment.

## Background

Among all bladder tumors, 70–75% are non-muscle-invasive (NMIBC: 70% Ta, 20% T1, 10% Tis) at first evaluation. NMIBC is a heterogeneous group composed of stages Ta (non-invasive papillary carcinoma), T1 (sub-epithelial connective tissue invasive carcinoma) and CIS (Tis Carcinoma in situ), and various grades including well (G1) moderately (G2) and poor (G3) differentiated tumors. Many large cohorts studies analyzed the long-term survival of NMIBC patients in general after transurethral resection (TUR) and intravesical chemotherapy [1]. Survival range varies between studies but are mostly higher than in muscle-invasive bladder cancer (MIBC), high grade (HG) Ta/T1 disease represents about 5 to 23% of NMIBC at first diagnosis, with a steady increase in incidence. It is considered as a highly malignant tumor with a variable and unpredictable biology as well as a critical aspect of management. Most of NMIBC cases are treated by TUR of the tumor, but about 50–70% undergoes recurrence within 2 years and 10–30% progresses to muscle-invasive disease [1]. The most accepted predictors of progression in these patients are tumor size, multifocality, presence of CIS and vascular invasion [2]. According to the European Organization for Research and Treatment of Cancer (EORTC) classification, any T1, HG/G3, CIS, recurrent multifocal and larger than 3 cm Ta G1-G2/Low grade (LG), are considered bladder cancer (BC) at high risk of progression to muscle-invasive disease [1, 3]. However, these features may not accurately reflect clinical outcome and only partially address a reliable risk-adjusted treatment planning. In the era of precision medicine, many efforts have been made to increase knowledge about disease biology, to find reliable biomarkers associated with progression in BC [3].

MicroRNA (miRNAs), short noncoding RNAs regulators of gene expression [4], are also present in several body fluids such as serum, plasma and urine, with a high degree of stability indicating their extensive potential as biomarkers [5, 6]. Recent literature has highlighted the dysregulation of several miRNAs, among them *let-7c* cluster, in BC tissue as well as in urine and blood, suggesting their potential role as diagnostic and prognostic biomarkers in BC patients [7–12].

*Let-7c* belongs to the *let-7* miRNA family that plays an important role in several cellular processes, and it is widely viewed as a tumor suppressor miRNA [13, 14]. *Let-7c* coding sequence, organized in a cluster with *miR-99a* and *miR-125b*, is localized on chromosome 21 and resides within intron 6 of the poorly characterized and recently named *LINC00478* gene [15]. Consistent with its tumor suppressor activity, *let-7c* is downregulated in many cancer, including BC, and has been related to tumor progression [16–18].

Here, we assessed the impact on Progression Free Survival (PFS) probabilities of urinary *let-7c* cluster expression to evaluate its suitability as potential non-invasive biomarker associated with NMIBC patients at higher risk of progression to muscle-invasive disease.

## Methods

### Patients and specimens

From January 2015 to March 2018, 57 urine and 49 neoplastic paired tissue samples were prospectively collected from TUR of HG-NMIBC patients at the “Regina Elena” National Cancer Institute. For 8 patients the tissue sampling was not available for experimental purposes. Twenty urine and ten bladder tissue samples were collected as healthy controls (HC), matched for age, sex, smoking habit and comorbidities. All patients received Bacillus Calmette-Guerin (BCG) induction and maintenance for a minimum period of 1 year according to Kamat’s protocol [19].

Urine cytology and cystoscopy were performed at 3 months’ intervals for the first two years and at 6 months’ intervals thereafter. A whole-body CT scan was performed yearly. TUR was performed in case of BC recurrence. Disease progression was defined as evidence of muscle-invasion at any subsequent TUR or evidence of metastatic spread at conventional imaging.

### Urine and tissue samples processing and RNA extraction

Urine was analyzed from each HC recruited at the same Institute from individuals seeking a routine health check-up, with no evidence of disease and with age-, sex- and ethnicity-matched to the patients. Urine samples obtained immediately before TUR procedure were analyzed from BC patients. Patients with positive urine culture and/or leukocyturia were excluded from the study. Normal tissue samples were obtained from bio-bank specimens of patients who underwent radical cystectomy (RC) [20].

Urine samples were collected in sterile recipient, stored at 4 °C, then centrifuged at 4.500 rpm for 30 min at 4 °C, followed by centrifugation at 8.900 rpm for 5 min to separate the supernatant from the urinary cell sediment. Urine supernatant was aliquoted in 2 mL-cryovials and stored at − 80 °C. After surgery, BC tissue samples were collected and stored in liquid nitrogen.

Total RNA was extracted from urine supernatants (starting from 2 mL) using the Urine cell free circulating RNA purification midi kit (#57000, Norgen Biotek, Canada). During extraction we added spike-in mix of non-human synthetic miRNAs (RNA spike-in mix: UniSp2, UniSp4 and UniSp5; #203203, Exiqon) into the lysis buffer before incorporating the urine sample. Frozen tissue specimens were homogenized and total RNA was obtained using Animal Tissues RNA Purification Kit (#25700, Norgen Biotek, Canada). RNA quantification from tissues was done by NanoDrop ND-1000 spectrophotometer (Thermo Fisher Scientific, Wilmington, DE U.S.A.).

### Cell culture and RNA extraction

RT-112 and T24 BC cell lines were kindly provided by Prof. Claudio Sette (“Università Cattolica del Sacro Cuore”, Rome, Italy). The RT-4 cell line was purchased from ATCC (Manassas, VA, USA). Human RT-4, RT-112 and T24 BC cell lines corresponding to LG-NMIBC, HG-NMIBC and HG-MIBC cell phenotype respectively, were grown in L-Glutamine-RPMI-1640 with 10% fetal bovine serum (Gibco®-Thermo Fisher), penicillin/streptomycin, gentamicin and MEM non-essential aminoacids (Sigma-Aldrich) at 37 °C in 5% CO2. Total RNA extraction from the culture medium (extracellular fraction), starting from 400 μL,was performed using the miRNeasy Serum/Plasma kit Adavanced (#217204, Qiagen). As technical control, we added spike-in mix of non-human synthetic miRNAs (RNA spike-in mix: UniSp2, UniSp4 and UniSp5; #203203, Exiqon) into the lysis buffer, before adding it to the supernatant sample. For culture medium collection, cells were seeded and after 24 h the medium was replaced with Serum-Free Medium (SFM). Culture medium was centrifuged at 2000 x g for 10 min, filtered using 0.22 μm polyether-sulfonate low-protein binding filters and aliquoted. Total RNA was isolated from cells (intracellular fraction) after 24 h from SFM (along with the cell supernatant collection). Cell pellet from BC cells was lysed in TRIsure™ (Bioline, Meridian Bioscience) for total RNA extraction. RNA quantification from cells was done by NanoDrop ND-1000 spectrophotometer (Thermo Fisher Scientific, Wilmington, DE U.S.A.).

### Complementary DNA (cDNA) synthesis and qRT-PCR

RNA reverse transcription (starting from 4 μL of RNA from urine and culture medium or 20 ng of tissue and cell RNA) from urine, tissues, cells and culture medium was performed using the Universal cDNA synthesis kit II (#203301, Exiqon, Denmark), including in the reaction spike-in Unisp6, to control cDNA synthesis reaction quality.

Mature cell-free (urine and culture medium) and tissue miRNAs quantification was performed by miRNA-specific LNA™-based system using SYBR® Green (miRCURY LNA™ Universal RT micro-RNA PCR; Exiqon # 203301, Vedbaek- Denmark). For qRT-PCR, cDNA was diluted 1:40 in nuclease-free water. ROX passive reference dye was added in diluted cDNA samples to obtain a robust read over the entire array of wells (ROX, Thermo Scientific, Waltham, Massachusetts, USA). The mix of qRT-PCR was composed of 5 μL ExiLENT SYBR Green Mastermix, 1 μL miRNA probe specific for hsa-let-7c-5p (LNA UniRT primer mix #204767, Exiqon), hsa-miR-99a-5p (LNA UniRT primer mix #205713, Exiqon) and hsa-miR-125b-5p (LNA UniRT primer mix #204521, Exiqon) miRNAs and 4 μL cDNA. QRT-PCR was performed using the ABI 7900 Real Time PCR System and SDS 2.2.2 software (Applied Biosystems, Foster City, CA). All the samples were tested in triplicate. We normalized urinary and culture medium miRNA levels with the expression of UniSP2 synthetic spike-in (UniSP2 LNA control primer set UniRT, #203950, Exiqon), according to previous report [21]. MiRNAs expression levels from tissue and cells were normalized using U6 snRNA (U6 snRNA LNA primer set UniRT, #203907, Exiqon). Relative quantification of miRNAs expression was determined using the comparative threshold cycle (ΔCt) method. MiRNA expression levels from culture medium was additionally normalized with the cell count.

### Statistical analysis

Descriptive statistics were used to summarize study data. The association between clinical variables and miRNA levels was tested by the Pearson Chi-Square test or Fisher’s Exact test, when appropriate. Differences of urinary and tissue miRNA levels between neoplastic and HC groups were compared using the non-parametric Mann-Whitney U test. MiRNAs expression levels were plotted into box-plots. Receiver operative characteristics (ROC) curves were used to identify the most informative miRNA expression thresholds predicting PFS. The prognostic role of miRNA cluster was tested with the Kaplan-Meier method. Survival rates were computed at 6, 12, 18 and 24 months after surgery, and the log-rank test was applied to assess statistical significance between the two groups. Univariable and multivariable Cox regression analyses were performed to identify predictors of PFS probabilities. A nomogram predicting disease progression risk was built. The discrimination accuracy was measured by concordance index (C-index). Calibration plot was generated with 200 bootstrap resampling. A decision curve analysis (DCA) was performed to assess the net benefit of the predictive model. Statistical analysis was performed with the Statistical Package for Social Science (SPSS Inc., v 21.0, Chicago, IL, USA), MedCalc (v. 14.10.2) and with STATA (Stata Statistical Software: Release 15. College Station, TX: StataCorp LLC).

## Results

### Expression levels of urinary let-7c/miR-99a/miR-125b cluster in HG-NMIBC patients

**Fig. 1:**
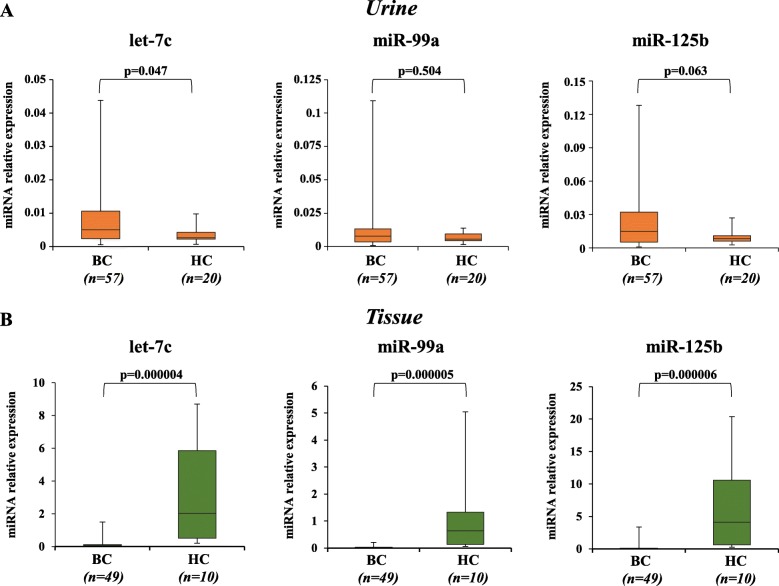
Expression levels of urinary and tissue *let-7c* miRNA cluster in a cohort of HG-NMIBC patients. Box-Plot diagrams of relative miRNA expression levels, performed by qRT-PCR in urine supernatant (**a**) and in tissue (**b**). Boxes define the 25th and 75th percentiles; the horizontal lines into within the boxes indicates the median, and bars define the minimum and maximum values. Relative miRNAs expression was normalized to Unisp2 spike-in for urine and U6 snRNA for tissue and calculated using the comparative ∆Ct method. *P*-value was determined using the Mann-Whitney rank sum test

To investigate the diagnostic role of cell-free urinary *let-7c/miR-99a/miR-125b* cluster in BC we assessed, by qRT-PCR, their expression levels in a selected cohort of HG Ta/T1 NMIBC which includes newly diagnosed and first recurrence patient samples (*n* = 57), compared to urine *let-7c* cluster expression levels of non-tumor HC (*n* = 20) matched for age and sex. Demographic, clinical and pathologic features of the cohort and general characteristics of HC are reported in Table 1. As shown in Fig. 1 panel A, HG-NMIBC patients displayed a significant up-regulation of urinary *let-7c* (+ 2.57 fold; *p* = 0.047), comparable levels of *miR-99a* and a trend toward significantly higher levels of *miR-125b* when compared to HC. We also evaluated the cluster expression levels in paired tissue (*n* = 49) of HG-NMIBC patients, compared to age- and sex- matched non-tumor bladder tissues of HC (*n* = 10). The results showed that the expression levels of the cluster were significantly decreased in tumor tissues compared to HC (*let-7c*, − 27.75 fold, *p* < 0.001; *miR-99a*, − 50.40 fold, *p* < 0.001; *miR-125b*, − 27.91 fold, *p* < 0.001; Fig. 1 panel B).

**Table 1:** Demographic, clinical and pathologic features of patients and general characteristics of healthy controls

Clinical features of patients	N or Mean (SD or %)	
Age	68.5 (11.4)	
Gender		
Male	51 (89.4)	
Female	6 (10.6)	
Disease history		
Primary	31 (54.4)	
Recurrent	26 (45.6)	
Smokers	37 (64.9)	
Diabetes	10 (17.5)	
Hypertension	34 (59.7)	
Stage		
Ta	35 (61.4)	
T1	22 (38.6)	
Multifocality		
Not	16 (28.1)	
Yes	41 (71.9)	
Tumor size > 3 cm	27 (47.4)	
Carcinoma in situ	4 (7%)	
EORTC score for progression		
Mean value	9.8 (4.9)	
Risk class 1 (2–6)	15 (26.4)	
Risk class 2 (7–13)	29 (50.8)	
Risk class 3 (14–23)	13 (22.8)	
Characteristics of healthy controls	**Urine Samples**	**Tissue Samples**
	**N or Mean (SD or %)**	
Total Number	20	10
Age (years)	62.2 (10.1)	69.8 (11)
Gender		
Male	17 (85)	7 (70)
Female	3 (15)	3 (30)

*EORTC* European Organization for Research and Treatment of Cancer

### Let-7c cluster expression levels and clinic-pathological features of HG-NMIBC patients

**Fig. 2:**
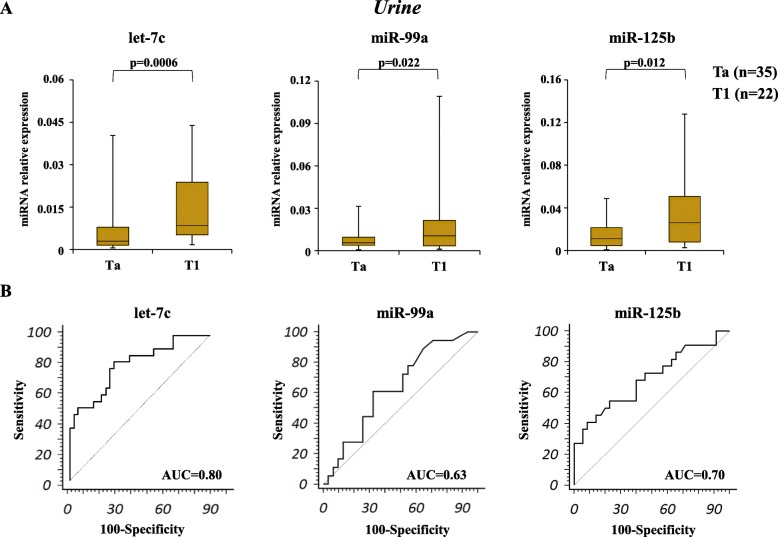
Association analysis between urinary *let-7c* cluster and tumor stage. **a** Box-plot diagrams of urinary *let-7c* cluster stratified into pathological Ta and T1 stages. Relative expression levels were normalized to Unisp2 spike-in and calculated using the comparative ΔCt method. P-value were determined using the Mann-Whitney rank sum test. Boxes define the 25th and 75th percentiles; the horizontal lines into in the boxes indicates the median, and bars define the minimum and maximum values. **b** ROC curves plotted for diagnostic potential and discriminatory accuracy of urinary *let-7c* cluster to distinguish T1 from Ta stage. The corresponding Area Under the Curve (AUC) values are reported

We evaluated the relationship between urinary and tissue expression levels of the *let-7c* cluster and the clinic-pathological parameters of the HG-NMIBC patients (Table 2). The results revealed a significant association between urinary *let-7c* cluster and tumor stage. In particular, higher expression of urinary *let-7c* and, to a lesser extent, of *miR-99a/−125b* were found in patients with T1 stage, compared to Ta stage (*let-7c*, + 2.46 fold, *p* = 0.0006; *miR-99a*, + 1.87 fold, *p* = 0.022; *miR-125b*, + 2.73 fold, *p* = 0.012; Fig. 2 panel A). In contrast, comparable expression of *let-7c* miRNA cluster in tumor tissues was found in Ta and T1 stage disease (*p* > 0.05; Figure S1 panel A). Next, we evaluated, by ROC curves analysis, the diagnostic performance and accuracy of urinary *let-7c* cluster to discriminate the tumor stage in HG-NMIBC patients. We found that urinary *let-7c* expression levels with an area under the curve (AUC) value of 0.80 (95% C-index = 0.670–0.892) were robust in discriminating T1 from Ta stage. In contrast, our analysis indicates a lower diagnostic performance for the other members of the cluster (*miR-99a*: AUC = 0.635, miR-125b: AUC = 0.691, Fig. 2 panel B). As expected, no substantial diagnostic performance was observed, by ROC curves, in paired tissues of the entire *let-7c* cluster (Figure S1 panel B; *let-7c* and *miR-99a*: AUC 0.60; miR-125b: 0.68 respectively). These data suggest that urinary *let-7c* cluster could be a reliable biomarker to distinguish T1 from the Ta stage in HG-NMIBC. The levels of *let-7c* cluster were not significantly associated with age, sex, tumor history, tumor size, smoking status, diabetes and hypertension (Table 2).

**Table 2:** Relationship between let-7c cluster and clinic-pathological features of NMIBC patients

	*Urine*					*Tissue*		
Variables	Cases N.	*p*-value	*p*-value	*p*-value	Cases N.	*p*-value	*p*-value	*p*-value
		let-7c	miR-99a	miR-125b		let-7c	miR-99a	miR-125b
Total Number	57				49			
Age (years)								
> 70	28	0.544	0.338	0.503	26	0.968	0.952	0.412
≤ 70	29				23			
Gender								
Male	50	0.589	0.712	0.934	44	0.298	0.876	0.419
Female	7				5			
TNM stage/HG								
T1	22	0.0006***	0.022*	0.012*	18	0.252	0.454	0.125
Ta	35				31			
Tumor size - diameter (cm)								
> 3	27	0.362	0.725	0.701	24	0.150	0.904	0.424
≤ 3	30				25			
Smoking status								
Yes	37	0.160	0.493	0.124	33	0.551	0.348	0.881
No	20				16			
History								
Newly diagnosed	31	0.956	0.744	0.402	26	0.768	0.231	0.573
Recurrent	26				23			
Diabetes								
Yes	10	0.629	0.314	0.675	9	0.737	0.179	0.918
No	47				40			
Hypertension								
Yes	34	0.425	0.290	0.935	32	0.065	0.401	0.721
No	23				17

### Evaluation of let-7c cluster expression levels in the extracellular fraction of BC cell lines

**Fig. 3:**
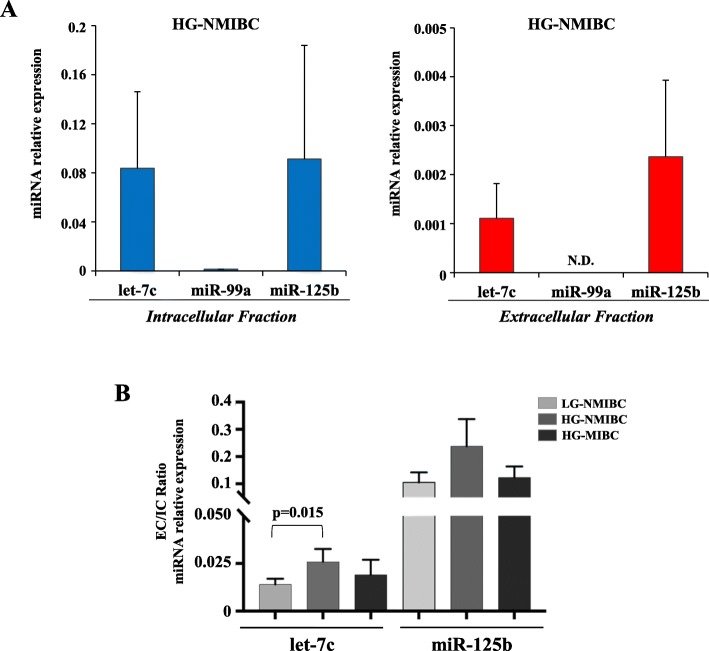
Analysis of *let-7c* miRNA cluster levels in BC cell lines. **a** Levels of *let-7c* cluster analyzed by qRT-PCR in intracellular (cells) and extracellular (culture medium) fractions of high grade (HG) NMIBC cells (RT-112). Relative expression was normalized with U6 snRNA and Unisp2 spike-in for intracellular and extracellular fraction respectively, and calculated using the comparative ∆Ct method. The extracellular values were normalized also to the cell count. N.D. indicates not determined miR-99a levels. **b** qRT-PCR of *let-7c *cluster of extracellular versus intracellular fraction (EC/IC ratio) in low grade (LG) NMIBC (RT-4), HG-NMIBC (RT-112) and HG-MIBC (T24) cells. Data are the average of three independent experiments; error bars indicate SEM. P-value was determined using the Mann-Whitney rank sum test

Cultured BC cells were used to investigate *let-7c* cluster expression levels in intracellular (cells) and extracellular (culture medium) fractions of BC cell line representative of the HG-NMIBC phenotype. We detected *let-7c* and *miR-125b*, but not *miR-99a*, both in intracellular and extracellular fractions although, as expected, with a relatively lower baseline amount in the extracellular fraction (Fig. 3 panel A). Next, we evaluated the extracellular to intracellular ratio of *let-7c* and *miR-125b* levels, comparing BC cells representative of different grade and invasiveness capability. Interestingly, we observed a higher ratio for *let-7c* in HG-NMIBC (RT-112) cells compared to the LG-NMIBC (RT-4) cell line, while no differences were detected compared to HG-MIBC (T24; + 2.61 fold, *p* = 0.015; Fig. 3 panel B).

### Urinary let-7c cluster as predictor of PFS probability

**Fig. 4:**
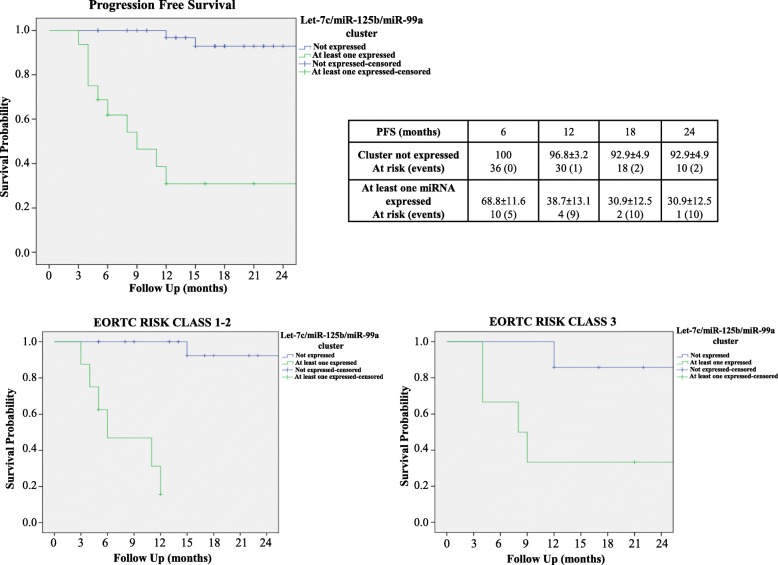
PFS probability in NMIBC according to urinary *let-7c* cluster expression. Log-Rank Curves showing PFS probability of patients with NMIBC according to the expression of *let-7c* cluster in urine and after stratifying for EORTC Risk Class

**Fig. 5:**
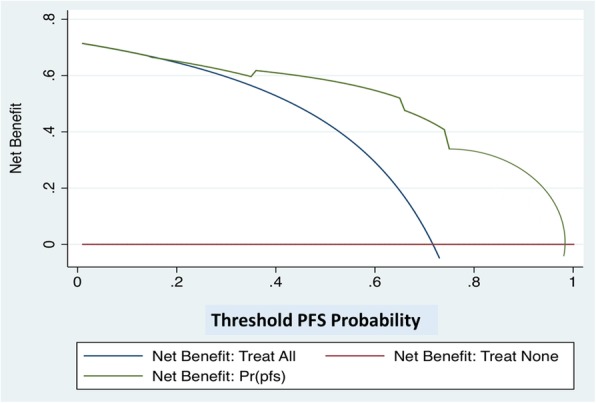
Decision Curve Analysis. DCA (Decision Curve Analysis) providing the benefit of using the nomogram including *let-7c* cluster expression in urine and the EORTC Score. The net benefit was evident for all the patients with a Progression Free Survival PFS probability Pr (pfs) > 20%

On ROC analysis, the most informative urinary levels cut-off values to discriminate PFS probabilities were 0.010 (AUC = 0.76), 0.035 (AUC = 0.77) and 0.020 (AUC 0.67) for *let-7c*, *miR-125b and miR-99a*, respectively (Figure S2). At a median follow up of 17 months (Interquartile range- IQR 12.5–22), 12 patients (21%) experienced disease progression. Upon univariable Cox regression analysis, lower EORTC scores and downregulation of the entire cluster were significant predictors of PFS probability (*p*=0.049 and *p*< 0.001 respectively; Table 3). Kaplan Meier analysis indicated that downregulation of the entire cluster was associated with significantly higher PFS even after stratifying for EORTC Risk Class 2 or 3 (both log rank *p* < 0.001, Fig. 4). Multivariable Cox regression analysis revealed that, only the cluster expression was an independent predictor of disease progression (HR 0.07; [0.02–0.26] *p* < 0.001; Table 3). The developed nomogram had a high predictive accuracy (C-index = 0.87) and was perfectly calibrated (Figure S3 panel A and B). On DCA, the net benefit of using the model was evident for all patients with a PFS probability > 20% (Fig. 5).

**Table 3:** Univariable and Multivariable Cox Regression Analysis for PFS

**Univariable Analysis**			
Variables	HR	CI	*p*-value
Age (continuous)	0.97	0.93–1.01	0.24
Gender	1.11	0.52–2.35	0.78
Smoke	1.07	0.63–1.81	0.80
Diabetes	0.53	0.12–2.35	0.40
Hypertension	0.46	0.16–1.35	0.16
pH	0.51	0.14–1.85	0.30
EORTC Score (Continuous)	1.14	1.00–1.29	0.049
Cluster	16.49	4.5–60.27	< 0.001
**Multivariable Analysis**			
Variables	HR	CI	*p*-value
EORTC Score (Continuous)	1.05	0.94–1.17	0.40
Cluster	0.07	0.02–0.26	< 0.001

*PFS* Progression Free Survival, *HR* hazard ratio, *CI* Confidence Intervals, *EORTC* European Organization for Research and Treatment of Cancer

## Discussion

Pathological and clinical features of BC, such as the tumor stage/grade, metastasis, recurrence and chemo-sensitivity are well known predictors of cancer specific survival [3]. Since HG-NMIBC patients display a non-negligible risk of recurrence and progression, BCG bladder instillation (induction and maintenance) is recommended due to its proven efficacy on reducing both recurrence and progression rates [3]. A recent EORTC study confirmed the role and the optimal schedule of BCG treatment following TUR [1]. Up to now, given the high risk of disease progression in BCG-failure patients, RC has been the gold standard treatment for these patients [3]. Early detection of patients with NMIBC at higher risk of progression by a non-invasive molecular marker, avoiding unsuccessful BCG treatment, and early identification of patients who may really benefit from an early RC, are of critical importance especially with regards to precision medicine.

Analysis of miRNAs seems to be the most promising strategy for the development of non-invasive early diagnostic/prognostic biomarkers because they are highly stable within urine, are more resistant against nuclease degradation and require little handling care [5–7].

*Let-7c* miRNA is widely accepted as a tumor suppressor miRNA involved in the regulation of oncogenic pathways and downregulated in many types of tumor tissue, including BC [13–18]. In line with *let-7c* tumor-suppressor role, our analysis of the cluster expression levels in HG-NMIBC patient’s tissue showed that *let-7c*, as well as *miR-99a* and *miR-125b*, were highly downregulated compared to normal tissue samples. In urine, *let-7c,* as well as the other *let-7c* cluster members, *miR-99a* and *miR-125b* has been found down-regulated in BC versus HC [11, 22, 23]. In the current study, we investigated the potential value of urinary *let-7c* cluster as non-invasive diagnostic/prognostic biomarker of HG-NMIBC patients. Our data showed a significant upregulation of urinary *let-7c* cluster in BC patients’ samples compared to HC. This observation is in contrast with previous findings showing decreased levels of urinary *let-7c* cluster in BC patients’ samples [11, 22, 23].

Potential reasons for divergences with previous studies may resides in the several challenges related to studying circulating miRNAs, especially the urinary ones. Among these a high degree of inter-individual variability of extracellular miRNAs levels [6], and technical sources of variation between studies (e.g., the RNA extraction method, the normalization, the technological platforms used etc.). For these reasons, to avoid technical biases, we maintained constant sample processing through the whole study. Moreover, it has to be considered that the debate about the relative contribution of organs and/or tissue to miRNA in biological fluids is still open since detailed knowledge of molecular mechanisms governing miRNA release from normal and tumor tissues is still lacking [6, 24, 25]. However, several authors in previous reports have shown opposite miRNA expression profile in bio-fluids versus matched tumour tissues [26, 27]. Our results showing that urinary *let-7c* expression is significantly upregulated and, coherently with its role of tumour suppressor, down-regulated in matched tissue samples compared to normal controls, fit with these cases. Moreover, we investigated the *let-7c* cluster levels in the extracellular fraction of BC cells characterized by the phenotype of HG-NMIBC.

It has been hypothesized that extracellular miRNAs may be released through both active and passive mechanisms [24]. In vitro studies also proved that miRNAs are exported into the cell supernatant [27]. Considering this, we wondered whether BC cells, with different grades and invasive power, could secrete *let-7c* cluster miRNAs in the cell medium, and examined its levels originating directly from the tumor. Interestingly, we found that *let-7c* cluster is detected in the culture medium (extracellular fraction) of BC cells with HG-NMIBC phenotype. Of note, by comparing the extra- to intra-cellular ratio between *let-7c* cluster levels in different grades and invasive power BC cells, we found *let-7c* ratio significantly higher in HG-NMIBC compared the other cell phenotypes. Accordingly, a previous study reported an enrichment of *let-7* miRNA family in the culture medium of gastric cells in relation with different tumorigenic and invasiveness propensities [27]. These data suggest that the increase of extracellular *let-7c* in HG-NMIBC cells could be a marker of disease at higher risk of progression to muscle-invasive BC. Moreover, the increased release of the tumor-suppressor *let-7c* in the culture medium found in the HG- compared to the LG-NMIBC cells could suggest a reduction of anti-cancer effect of *let-7c* in the cells, to maintain their oncogenic potential, as already observed in other tumor systems [27].

Several reports associate miRNA expression to cancer stage, also in BC [8, 11, 28]. In the present study we found a relevant association of increased urinary *let-7c* cluster levels in T1 stage disease. These data, even if provided by histological examination from TUR, suggests that the increased urinary *let-7c* cluster levels could early discriminate patients at higher risk of tumor progression as T1 HG-NMIBC. In matched tumour tissues, the T1 versus Ta tumour stages comparison did not lead to substantial statistically differences, thus, only the urinary miRNAs expression shows the capability of discriminating the higher and more advanced T1 stage with a greater risk of tumor progression in HG-NMIBC patients from the Ta stage. These data, in liquid biopsy vs tissue specimens, suggest the importance of *let-7c* cluster in view of the long-term goal to potentially reduce or avoid all forms of invasive procedures. Regarding the prognostic stratification of NMIBC patients, EORTC tables, based on the six most significant clinical and pathological features, are well-known tools to calculate the risk of disease progression in NMIBC patients [29]. Risk stratification by EORTC progression score was confirmed in many reports. May et al. reported, in a series of 521 RC performed for MIBC, that baseline high risk NMIBC had significantly worse cancer survival after RC when compared with baseline low-to-intermediate EORTC progression risk score patients [30]. In our study, upregulation of urinary *let-7c* cluster has an impact on PFS probabilities thus suggesting that its expression may improve prognosis estimation, identifying patients at risk of progression. EORTC score and *let-7c* cluster expression under the threshold value, determined in ROC curve analysis, were significant predictors of PFS on univariable Cox regression analysis. On multivariable analysis, miRNA profiling was the only independent predictor of PFS in HG-NMIBC patients. In fact, in both intermediate and high risk EORTC score cohorts, patients with urinary expression for at least one member of the cluster displayed worse PFS (both log rank *p* < 0.001). On DCA, the benefit of using this prognostic model was evident in patients displaying a PFS > 20%. This cohort essentially includes all cases where the clinical decision making process between conservative options (intravesical BCG) and RC is controversial, being those patients with a PFS probability < 20% with a significant risk of disease progression, where the effectiveness of conservative treatments should be carefully balanced with the significant risk of progression.

The limitations of our study are the small sample size, and the tumor heterogeneity and multifocality that may hide variability in miRNA expression. Therefore, external validation of these findings with larger prospective studies is mandatory to confirm findings and to consider the clinical application of miRNA assays for diagnostic and prognostic purposes in HG-NMIBC.

## Conclusions

Our study provides a proof for the potential use of cell-free miRNA biomarkers in clinical practice. We found that urinary expression of *let-7c* miRNA cluster could be a reliable non-invasive clinical tool to improve prognosis prediction of HG-NMIBC and to counsel patients according to an individual assessment of disease progression risk. Thus, from a practical point of view the importance of this potential assay is to intercept patients that undergo TUR of the tumor and are at risk of progression to address a more radical treatment.

## Supplementary information

**Additional file 1 **: **Figure S1.** Association analysis between tissue *let-7c* cluster and tumor stage.

**Additional file 2 **: **Figure S2. ***Let-7c* cluster cut-off values for PFS probability.

**Additional file 3 **: **Figure S3.** Predictive nomogram of 12-months and 24 months of PFS Probability.

## Data Availability

Data and materials are available for sharing if needed

## Abbreviations

AUC: Area Under Curve
BC: Bladder Cancer
BCG: Bacillus Calmette-Guerin
cDNA: Complementary DNA
CI: Interval of Confidence
C-index: Concordance Index
CIS: Carcinoma in Situ
Ct: Cycle threshold
DCA: Decision Curve Analysis
DCA: Decision Curve Analysis
EC: Extracellular
EORTC: European Organization for Research and Treatment of Cancer
HC: Healthy Controls
HG: High Grade
HR: Hazard Ratio
IC: Intracellular
IQR: Interquartile Range
LG: Low Grade
miRNA: microRNA
NMIBC: Non-Muscle-Invasive Bladder Cancer
PFS: Progression Free Survival
qRT-PCR: quantitative Real time-Polymerase Chain Reaction
RC: Radical Cystectomy
ROC: Receiver Operating Characteristic
SFM: Serum Free Medium
TUR: Transurethral Resection
