# Supplementary figures and images for: Urinary expression of let-7c cluster as non-invasive tool to assess the risk of disease progression in patients with high grade non-muscle invasive bladder Cancer: a pilot study
Source: J Exp Clin Cancer Res. 2020 Apr 17;39:68. doi: 10.1186/s13046-020-01550-w (PMC7164295; doi:10.1186/s13046-020-01550-w)

**A***Tissue*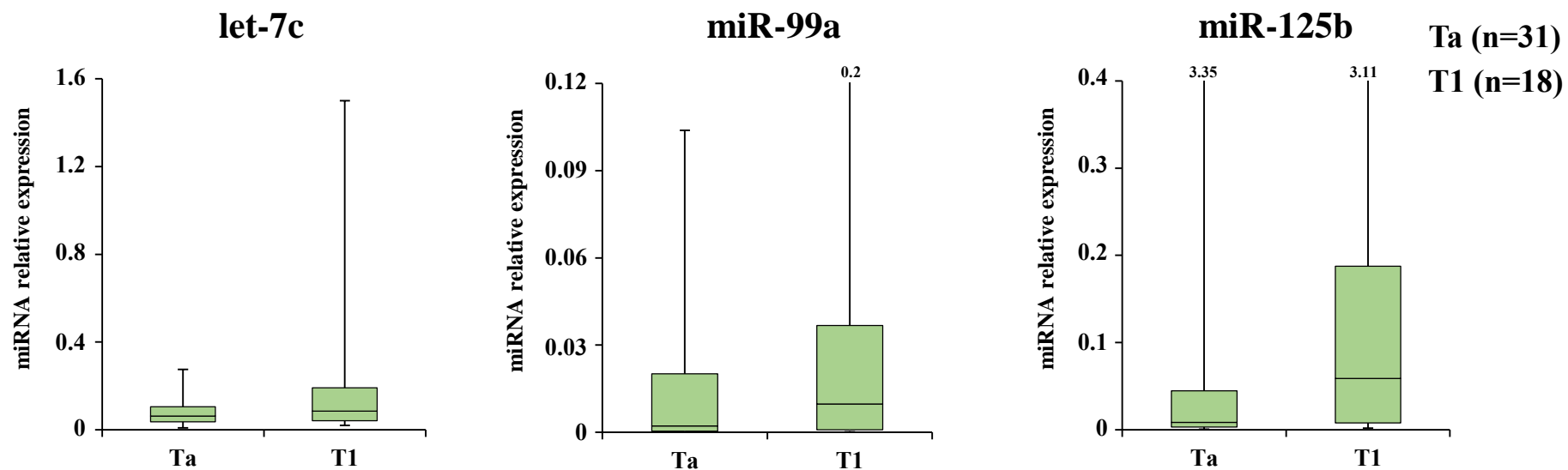**B**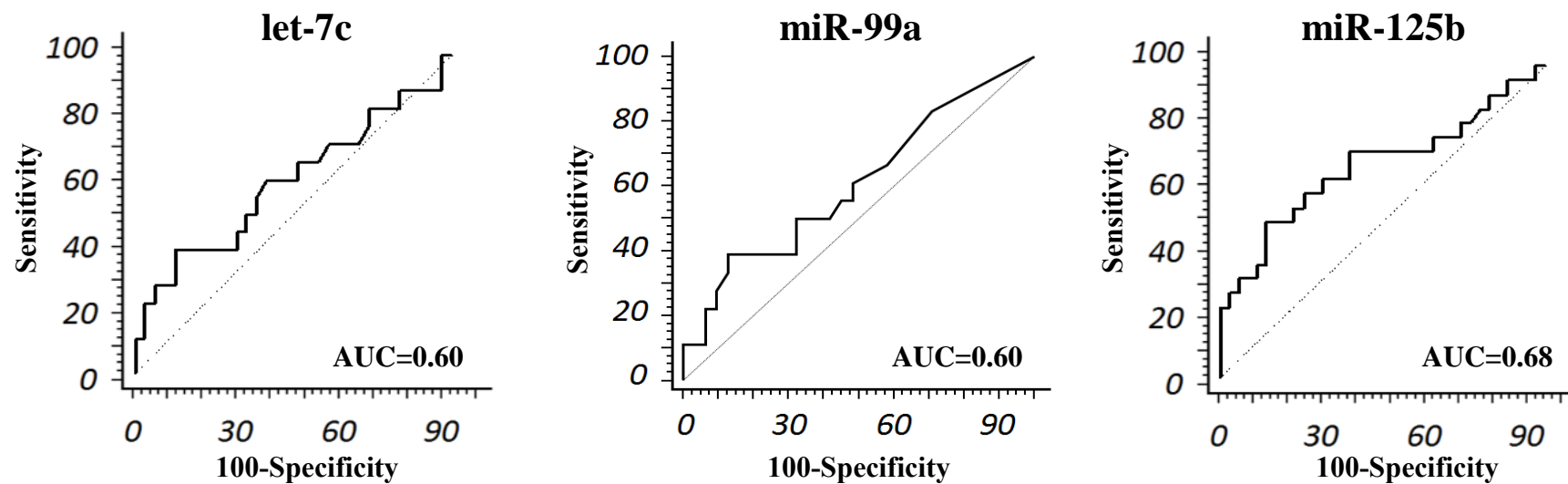

Supplement: Supplementary file 1 — Additional file 1 : Figure S1. Association analysis between tissue let-7c cluster and tumor stage. [file 13046_2020_1550_MOESM1_ESM.pdf]

**Figure S2**

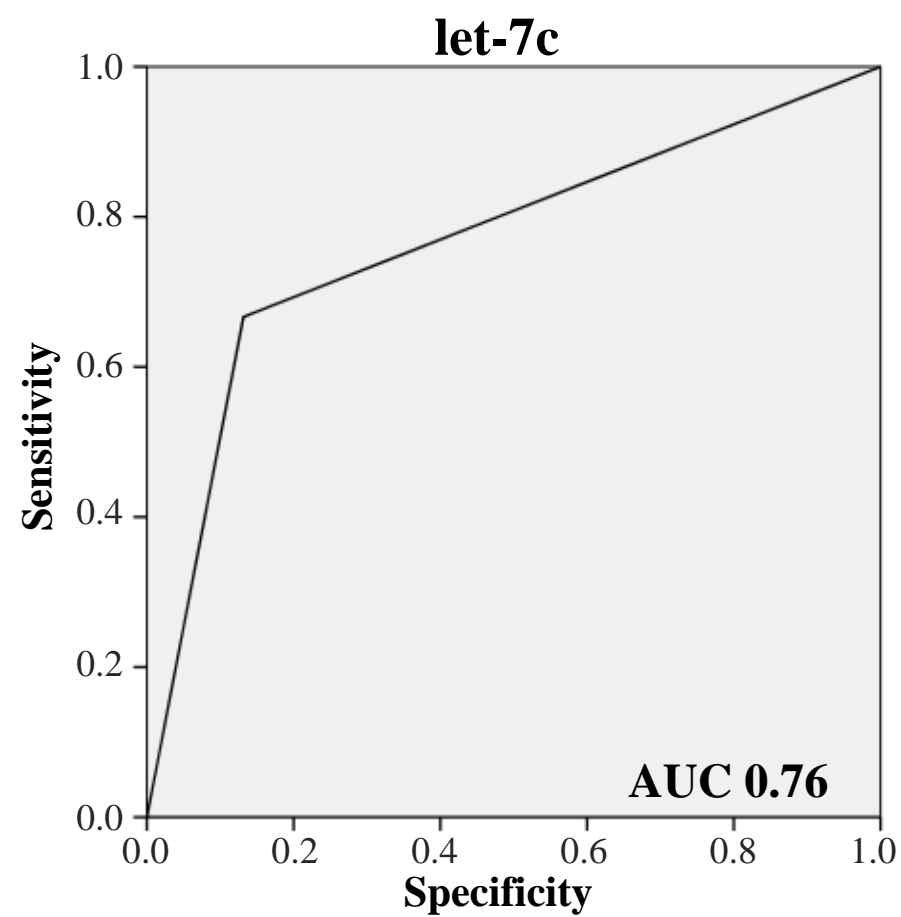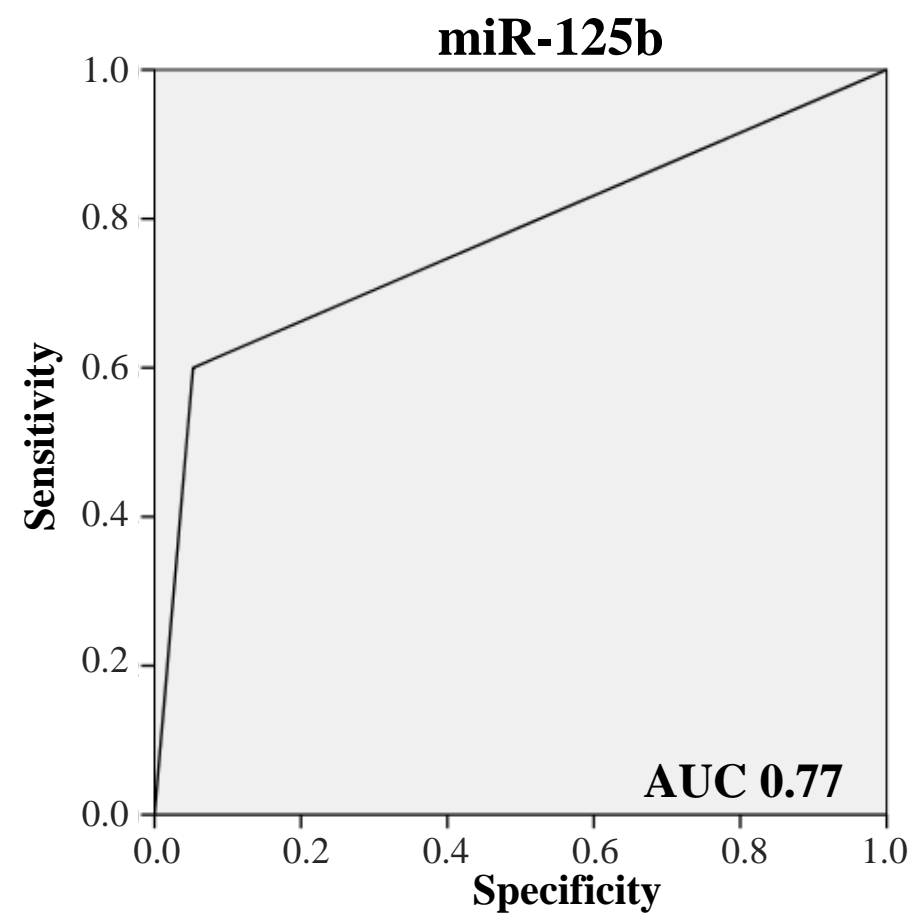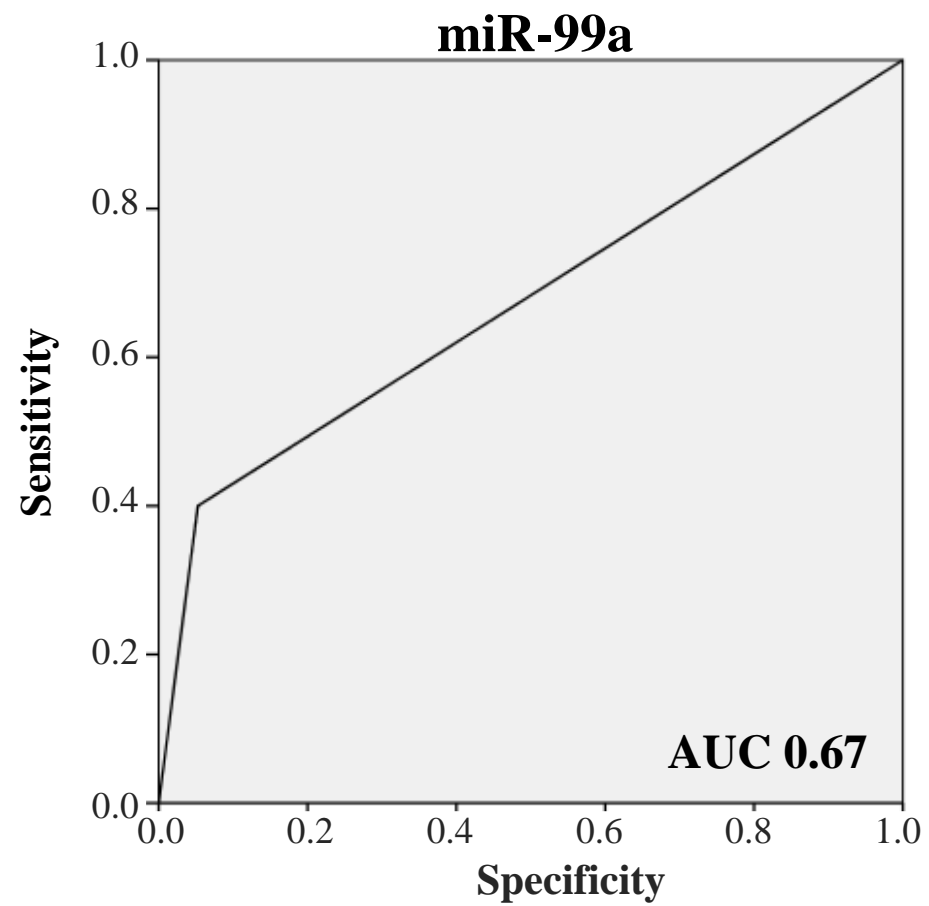

Supplement: Supplementary file 2 — Additional file 2 : Figure S2. Let-7c cluster cut-off values for PFS probability. [file 13046_2020_1550_MOESM2_ESM.pdf]

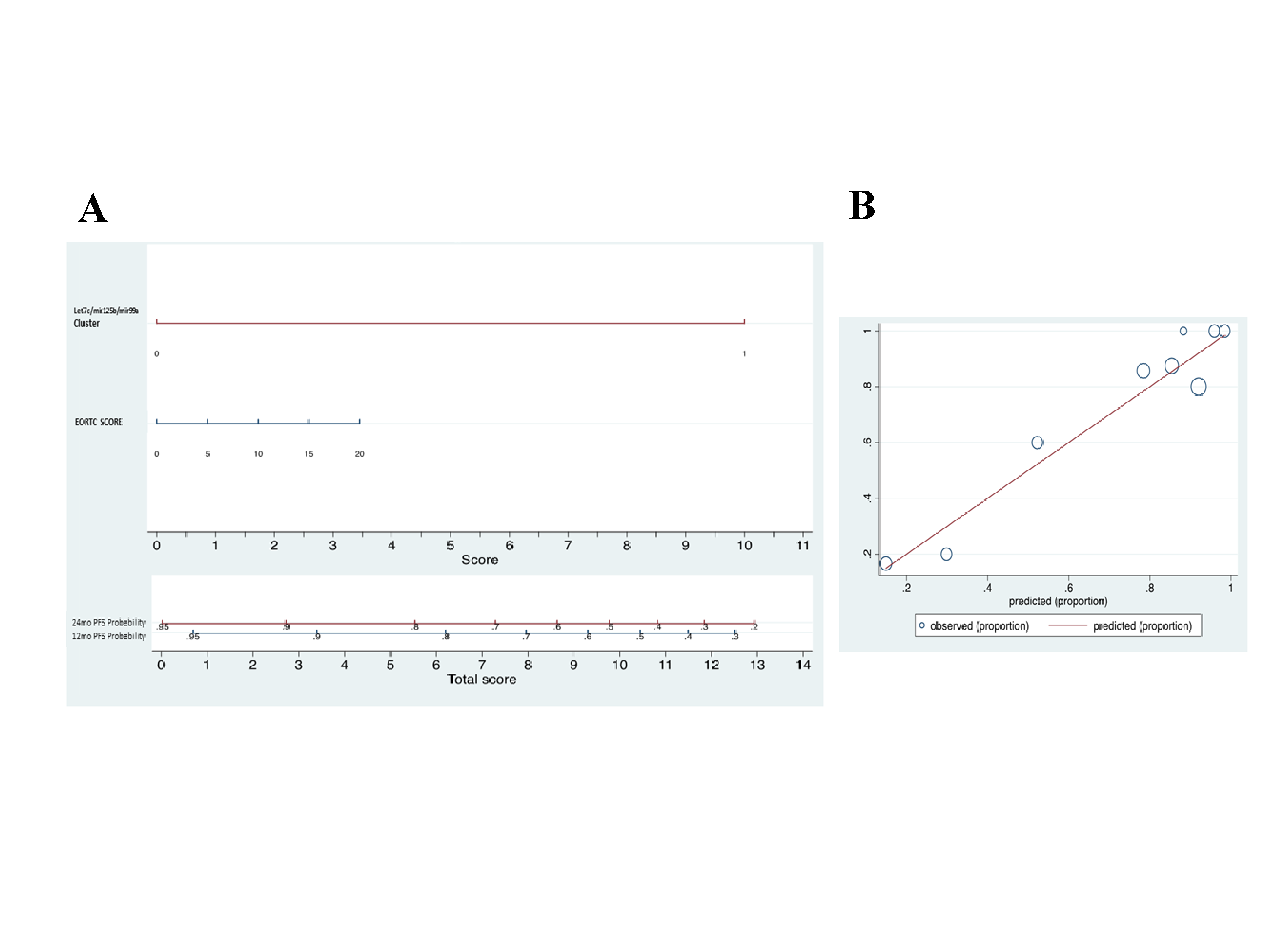

Supplement: Supplementary file 3 — Additional file 3 : Figure S3. Predictive nomogram of 12-months and 24 months of PFS Probability. [file 13046_2020_1550_MOESM3_ESM.tif]
